# Dengue virus susceptibility in novel immortalized myeloid cells

**DOI:** 10.1016/j.heliyon.2020.e05407

**Published:** 2020-11-03

**Authors:** Atsushi Yamanaka, Kazuo Miyazaki, Jun Shimizu, Satoru Senju

**Affiliations:** a Mahidol-Osaka Center for Infectious Diseases, Faculty of Tropical Medicine, Mahidol University, 420/6 Ratchawithi Road, Ratchathewi, Bangkok, 10400, Thailand; b Mahidol-Osaka Center for Infectious Diseases, Research Institute for Microbial Diseases, Osaka University, 3-1 Yamada-oka, Suita, Osaka, 565-0871, Japan; c MiCAN Technologies Inc., KKVP, 1-36 Goryo-ohara, Nishikyo-ku, Kyoto, 615-8245, Japan; d Department of Immunogenetics, Graduate School of Medical Sciences, Kumamoto University, 1-1-1 Honjo, Chuo-ku, Kumamoto, 860-8556, Japan

**Keywords:** Immunology, Infectious disease, Microbiology, Virology, Antibody, Dengue virus, Myeloid cell, iPS cell, Regenerative medicine, Antibody-dependent enhancement

## Abstract

Human dendritic cells (DCs) are the main target cells of dengue virus (DENV). Because humans injected with even a small volume of DENV from mosquito saliva display a high level of viremia, DCs are expected to be highly susceptible to DENV. In the present study, we assessed the efficiency of DENV infection using the novel immortalized human myeloid cell lines iPS-ML and iPS-DC. To prepare the DC-like myeloid cell line (iPS-DC), iPS-ML cells were cultured in the presence of IL-4 for 72 h. iPS-DC cells were the most susceptible to DENV, followed by iPS-ML, Vero and K562 cells. In contrast, the highest infective yield titer was observed in Vero cells. To investigate further uses of iPS-ML and iPS-DC, these cells were applied to an assay measuring antibody-dependent enhancement (ADE) activity in DENV infection. Serum samples collected from healthy Thai participants and mouse monoclonal antibodies displayed similar ADE activity patterns when examined with iPS-ML, iPS-DC, or K562 cells, the last of which are usually used in conventional ADE assays. Interestingly, iPS-ML cells showed greater susceptibility to ADE activity than iPS-DC and K562 cells. Here, we demonstrated the potential utility of the novel immortalized human myeloid cell lines iPS-ML and iPS-DC in future research on DENV.

## 1 Introduction

Dengue virus (DENV) has four genetically distinct serotypes (DENV-1, DENV-2, DENV-3 and DENV-4), which belong to the genus Flavivirus, family Flaviviridae [1]. DENV is one of the most important mosquito borne viruses and is the causative agent of dengue and severe dengue [2]. An estimated 390 million DENV infections occur annually in humans, with approximately 100 million infected individuals exhibiting clinical symptoms, such as high fever, muscle and joint pains, headache, rash etc. [2, 3]. Currently, the only commercially available dengue vaccine is Sanofi's Dengvaxia, which has been approved for use in several endemic countries in Asia, Europe and the Americas. Although Dengvaxia was partially effective in protecting seropositive vaccine recipients from severe diseases, there were some cases of severe/critical disease among younger seronegative vaccinees [4]. Eventually, the Sanofi company issued a warning that the incidence of severe disease might increase in seronegative populations, who were not previously infected with DENV, upon subsequent DENV infection following the vaccination [5]. Antibody-dependent enhancement (ADE), which enhances viral entry via Fc gamma receptors, is one of the pathogenic mechanisms increasing disease severity [6, 7]. Accordingly, for a dengue vaccine to be successful it must be safe and must not induce ADE. Therefore, before the dengue vaccine can be confidently administered to large populations worldwide, there is need of an appropriate antibody survey system to accurately and sensitively determine not only the seronegative/seropositive status, but also the functional antibody status (protective neutralization or pathogenic ADE) of potential vaccinees.

DENV, which is transmitted by infected mosquitoes, initially targets dendritic cells (DCs) in human skin [8, 9]. Infected DCs migrate to the lymph nodes and transmit DENV to immune cells, including monocytes and T-cells, during the process of antigen presentation [10]. Primary virus replication and dissemination into the blood and organs results in high levels of viremia (approximately 10^9^ cDNA Eq/ml) in humans [11]. Although the small volume of initially injected mosquito saliva contains fewer than 100 plaque forming units (PFU) of DENV [12], when the DENV becomes amplified, various clinical symptoms develop [13]. Therefore, as the initial target cells of DENV, DCs are critical for primary replication in the human epidermis/dermis during the early infection phase [14, 15]. Moreover, another group has demonstrated that primary DCs showed ADE in DENV infection [16]. Nonetheless, despite the high relevance of DCs for basic and applied dengue research [17], the preparation of DCs is very labor intensive, making it difficult to culture stable DCs in the laboratory [18].

In the present study, we assessed two novel immortalized human myeloid cell lines to determine whether they have susceptibility to DENV and thus could be useful in an antibody assay to detect ADE activity.

## 2 Materials and methods

### 2.1 Cells and viruses

Previously, Haruta et al. developed a human immortalized monocyte-like myeloid cell line, iPS-ML, derived from induced pluripotent stem (iPS) cells [19]. iPS-DC, a DC-like myeloid cell line, was differentiated from iPS-ML cells by incubation in the presence of IL-4 at a final concentration of 100 ng/ml at 37 °C for 3 days [19, 20]. Both iPS-ML and iPS-DC cells were cultivated in minimum essential medium (MEM) Alpha supplemented with 10% fetal bovine serum (FBS). African green monkey kidney Vero cells were cultivated in Eagle's MEM supplemented with 10% FBS and 60 μg/mL kanamycin [21]. Human erythroleukemia K562 cells were cultivated in RPMI 1640 medium supplemented with 10% FBS, 100 units/mL penicillin, and 100 μg/mL streptomycin [22]. All cell lines were cultivated in a humidified atmosphere of 5% CO_2_:95% air at 37 °C. Four previously described prototype DENVs (DENV-1, Mochizuki strain; DENV-2, New Guinea C strain; DENV-3, H87 strain; and DENV-4, H241 strain) [23] were used in this study. Viruses were harvested from the culture fluids of infected Vero cells.

### 2.2 Human sera and mouse monoclonal antibodies

Serum samples were collected from healthy Thai individuals who provided their written informed-consent before participation in the study. All participants completed a questionnaire survey, and none reported having experienced any symptoms of dengue or having been diagnosed with dengue fever or dengue hemorrhagic fever previously. The sera were heat-inactivated at 56 °C for 30 min. The study was conducted in accordance with the Declaration of Helsinki, and the protocol was approved by the Ethics Committee of the Faculty of Tropical Medicine of Mahidol University (Approval No. MUTM 2017-08-03). The mouse monoclonal antibody (D1-V-3H12) in an ascites form was previously generated from a mouse immunized with the DENV-1 Mochizuki strain [24]. D1-4G2 antibody (Envelope-specific, flavivirus group-crossreactive) was purchased from the American Type Culture Collection (Manassas, VA).

### 2.3 Inoculation of DENV into cells

iPS-ML, iPS-DC, Vero or K562 cells were prepared in poly-L lysine-coated 96-well microplates (1×10^5^ cells/well) and inoculated with DENV (DENV-1, DENV-2, DENV-3 or DENV-4) at 37 °C for 2 h. Two assay methods, based on (i) visualizing infected cells and (ii) measurement of the viral yield, were used to evaluate the susceptibility. (i) After 48–72 h, the cell layers were fixed with acetone–methanol (1:1) and immunostained with D1-4G2 antibody, using avidin-biotinylated peroxidase complex (ABC) reagent (Vector Laboratories, Burlingame, CA) or Alexa Fluor 488 (Invitrogen, Gaithersburg, MD), as described previously [22]. (ii) After 72–96 h, supernatants were harvested from the infected cells. The infective titers were determined on Vero cells by counting infectious foci after immunostaining with ABC reagent and expressed as focus-forming units (FFU).

### 2.4 Conventional neutralization test

The Vero cell plaque reduction neutralization tests of serum samples were performed using DENV-1 to DENV-4, as described previously [22]. Briefly, mixtures of the virus and two-fold serial dilutions of serum samples (starting from 1:10) were incubated at 4 °C overnight. Vero cell monolayers prepared in a 24-well microplate were inoculated with the virus–antibody mixture and incubated at 37 °C for 3 days. After fixation and immunostaining using 4G2 antibody, the plaques were counted. The neutralizing activities were expressed as percentages of plaque reduction calculated relative to the results for virus controls without test samples. The neutralizing antibody titers were expressed as the maximum serum dilution showing >75% plaque reduction (PRNT75).

### 2.5 Assay to detect ADE activity in serum samples and monoclonal antibodies

The infection-enhancing activities of the serum samples and monoclonal antibodies were measured using iPS-ML, iPS-DC or semi-adherent K562 cells, and expressed as the fold enhancement of infection. This assay was performed essentially following the previously described method [25]. Briefly, serial ten-fold dilutions of antibody specimens were mixed with DENV-4 preparation 1.0×10^3^ FFU in a poly-L lysine-coated 96-well microplate. After incubation at 37 °C for 2 h, iPS-ML, iPS-DC or K562 cells were added (1×10^5^ cells/well) and further incubated at 37 °C. After 72 h, the infective yield titers in the culture supernatant were determined by the focus-forming assay on Vero cells. The fold enhancement, representing ADE activity, was expressed in log10 as the increase in the yield of virus titer relative to the negative control, following the method used in our previous studies with a minor modification [24, 25].

### 2.6 Statistical analysis

The statistical significance of differences was evaluated with a Student's *t*-test. Probability (p) less than 0.05 was considered significant.

## 3 Results and discussion

**Figure 1:**
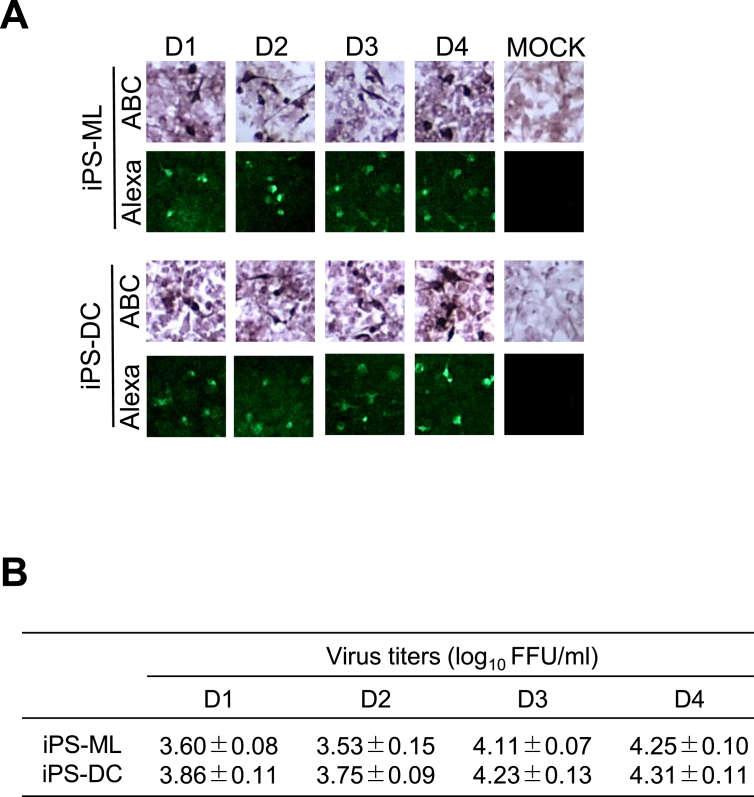
Infection with DENV-1–DENV-4 (A) Micrographs of iPS-ML and iPS-DC infected with DENV-1–DENV-4 (D1–D4) and MOCK. Dark (ABC) or fluorescent (Alexa) staining with Envelope-specific 4G2 antibody is the result of specific immunoreactivity of infected cells. Infected–iPS-ML and –iPS-DC cells cultured in semi-adhesive monolayer exhibited similar morphology with protrusions. (B) Infective yield titers in the supernatant. The virus titers (FFU/ml) in the supernatant harvested from infected–iPS-ML and –iPS-DC cells after 48 h were determined by the focus-forming assay on Vero cells. The values are expressed as mean ± SD that were obtained from two independent assays.

To investigate the susceptibility of iPS-ML and iPS-DC to DENVs, these cells were inoculated with DENV-1–4 at a multiplicity of infection (MOI) of 0.001, or mock infected (MOCK). After 48 h, the infected cell layers were immunostained with Envelope-specific 4G2 antibody. As shown in Figure 1A, both iPS-ML and iPS-DC cells were susceptible to all four serotypes of DENV with the infection rate of approximately 5–10 % irrespective of the four serotypes. However, as shown in the micrographs, these cells did not form foci, even when they formed a semi-adhesive monolayer. The virus titers in the supernatant collected from infected-cells at an MOI of 0.001 were shown in Figure 1B, suggesting that the yield levels obtained with DENV-3 and DENV-4 were higher than those with DENV-1 and DENV-2. Therefore, the virus yield in the culture supernatant harvested from the infected cells was used for further evaluation of the susceptibility of iPS-ML and iPS-DC.

**Figure 2:**
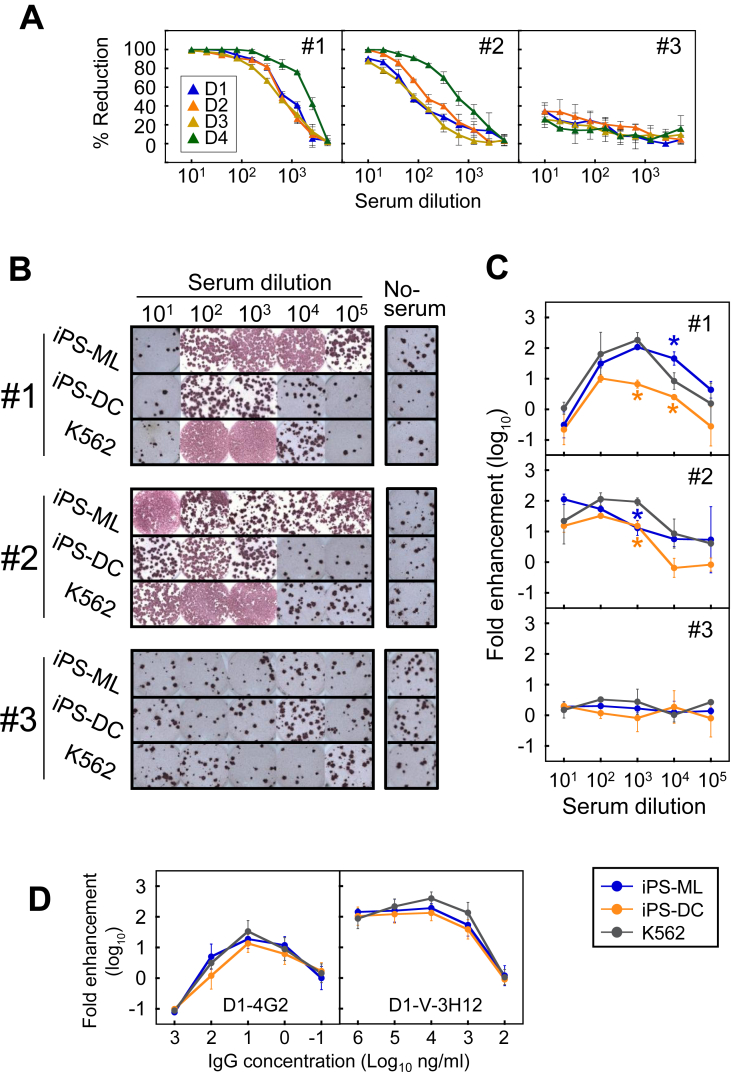
Application of iPS-ML and iPS-DC to ADE assay. (A) Neutralizing activities of three human sera against DENV-1–DENV-4 (D1–D4) were determined by a conventional neutralization test on Vero cells. (B) ADE activities in human sera from three individuals were detected on iPS-ML, iPS-DC and K562 cells. As a negative control, iPS-ML, iPS-DC or K562 cells were infected with DENV-4 in the absence of any sera (No-serum). The viral titer in the undiluted supernatant was determined by culture on Vero cells followed by immunostaining with ABC reagent. (C) Dose-dependent ADE activity curves of three human sera against DENV-4. Infective titers in the culture supernatant from Figure 2B were determined by focus-forming assay on Vero cells. ADE activity was expressed as the fold enhancement. (D) Dose-dependent ADE activity curves of mouse monoclonal antibodies (D1-4G2 and D1-V-3H12) against DENV-4. Data represent the averages of two independent assays + SD. Asterisks indicate significant differences (P < 0.05) from the fold enhancement obtained with K562 cells, which have often been used for measuring ADE activity.

To investigate the possible use of iPS-ML and iPS-DC as Fc gamma receptor-bearing cells, which have been shown to express three types of Fc gamma receptors (FcγRI, FcγRII and FcγRIII) [20], iPS-ML and iPS-DC cells were applied to an assay measuring ADE activity in DENV infection. Three serum samples (#1: female, 36 years old; #2: male, 32 years old; #3: male, 27 years old) collected from healthy Thai individuals were used as antibody specimens. A conventional neutralization test revealed that the PRNT75 titers against DENV-4 in samples #1 and #2 were 1:1280 and 1:160, respectively, and these values were 4–8-fold higher than those against the other serotypes (DENV-1–3) (Figure 2A). Thus, samples #1 and #2 were suspected to have been previously infected with or exposed to DENV-4. Therefore, their enhancing activities were investigated with a focus on DENV-4 (Figure 2B). In contrast, serum sample #3 showed no detectable neutralizing activity (<1:10) against any serotypes; sample #3 was thus used as a seronegative control. ADE activities against DENV-4 in human serum samples are visually shown in Figure 2B. Interestingly, iPS-ML showed greater susceptibility to ADE activity across a wide range of dilutions of samples #1 and #2 than did iPS-DC and K562 cells. On the other hand, no ADE activity was observed in #3 using any cells. These results indicate that other potential cofactor(s), in addition to Fc gamma receptors, may be involved in ADE; these factors will be investigated in a future study. Fold enhancement was calculated based on the virus yield titers obtained in Figure 2B, and the dose-dependent enhancing activity curves for each sample are shown in Figure 2C. Typical dose-dependent ADE activity patterns were observed in the seropositive samples (#1 and #2) displaying ADE activities against DENV-4 at multiple serum dilutions. The peaks of ADE activity in samples #1 and #2 were found at 10^2−3^ and 10^1−2^ serum dilutions, respectively, at subneutralizing doses observed in the ADE assay. Although several significant differences were observed, the dose-dependent curves obtained from human sera were relatively similar among the three cell lines. Moreover, the dose (antibody dilution)-response antibody activity curves obtained from mouse monoclonal antibodies (D1-V-3H12 and D1-4G2) were more similar among the three cell lines (Figure 2D). These suggest that iPS-ML and iPS-DC can be used in place of K562 cells for ADE assay in DENV infection.

**Figure 3:**
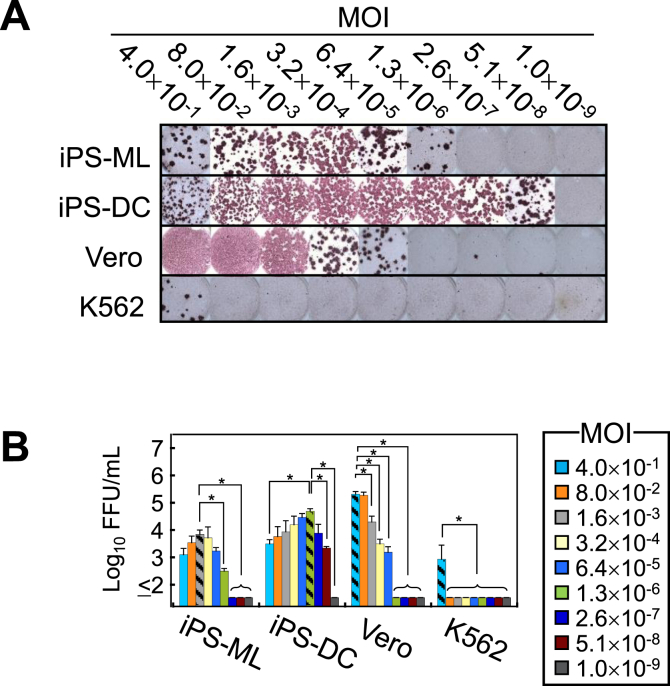
Susceptibility of iPS-ML and iPS-DC to DENV-4. (A) Comparison of susceptibility to DENV-4 in four cell lines. The viral titer in the undiluted supernatant was determined by culture on Vero cells followed by immunostaining with ABC reagent. (B) Comparison of the yield titers secreted from four infected cell lines. Infective titers in the culture supernatant harvested in Figure 3A were determined by focus-forming assay on Vero cells. Data represent the averages of two independent assays + SD. Asterisks indicate significant differences (P < 0.05) from the FFU/ml value corresponding to the highest yield titer (striped bars) obtained with the appropriate MOI.

To further evaluate susceptibility to DENV, serial five-fold dilutions of DENV-4 inoculum (starting from an MOI of 4.0 × 10^−1^) were inoculated onto iPS-ML, iPS-DC, Vero and K562 cells. The infective yield titers obtained from the supernatant 96 h after inoculation indicated that iPS-DC was the cell line most susceptible to DENV-4 (Figure 3A). Specifically, iPS-DC cells were susceptible to DENV-4 even at an MOI of 5.1 × 10^−8^. Susceptibility of iPS-ML was slightly higher than that of Vero cells. In contrast to the susceptibility, the highest yield titers displayed by iPS-DC and iPS-ML (4.9×10^4^ FFU/ml at an MOI of 1.3 × 10^−6^ and 7.2×10^3^ FFU/ml at an MOI of 1.6 × 10^−3^, respectively) were significantly lower (P < 0.05) than that by Vero cells (2.1×10^5^ FFU/ml at an MOI of 4 × 10^−1^) (striped bars in Figure 3B). Interestingly, the highest inoculum (MOI of 4 × 10^−1^) tended to produce a low level of virus yield from infected–iPS-DC and –iPS-ML. In particular, the yield titer obtained from iPS-DC infected with DENV-4 at an MOI of 4 × 10^−1^ was significantly lower (P < 0.05) than that at an MOI of 1.3 × 10^−6^. These results suggest a hypothesis that the high susceptibility of iPS-DC (and iPS-ML) to DENV might lead to excessive damage to cells or apoptosis following inoculation with a high-titer of virus [26], which might affect their capacity to yield progeny virus.

In conclusion, the present study has introduced and demonstrated that novel immortalized cells (iPS-ML and iPS-DC) can be applied to several DENV assays, which may contribute to elucidation of the pathogenic mechanisms during early infection [15]. In particular, iPS-DC is highly susceptible to DENV; thus this cell line may facilitate virus isolation from clinical samples containing very low viral titers due to delayed sample collection, which will be studied in the future. Further, we demonstrated that iPS-ML and iPS-DC could be useful to detect ADE of DENV infection, as a new system to measure ADE using human myeloid cell lines. iPS-ML and iPS-DC showed not only higher susceptibility to DENV than K562, but also a wider detection range of ADE activity, and thus these cells could be used in place of K562 cells, the Fc gamma receptor-bearing cells that have been generally used for the detection of ADE activity in DENV infection [22, 24, 25]. The present system could serve as an alternative ADE assay and might become necessary for further evaluation of ADE from a clinical perspective [4, 27].

## Declarations

### Author contribution statement

Atsushi Yamanaka: Conceived and designed the experiments; Performed the experiments; Analyzed and interpreted the data; Contributed reagents, materials, analysis tools or data; Wrote the paper.

Kazuo Miyazaki: Conceived and designed the experiments; Analyzed and interpreted the data; Contributed reagents, materials, analysis tools or data; Wrote the paper.

Jun Shimizu: Performed the experiments; Analyzed and interpreted the data; Contributed reagents, materials, analysis tools or data; Wrote the paper.

Satoru Senju: Contributed reagents, materials, analysis tools or data.

### Funding statement

This work was supported by the Japan Initiative for Global Research Network on Infectious Diseases (J-GRID) from the 10.13039/501100001700Ministry of Education, Culture, Sport, Science & Technology in Japan, and the 10.13039/100009619Japan Agency for Medical Research and Development (AMED) (JP18fm0108003).

### Competing interest statement

The authors declare no conflict of interest.

### Additional information

No additional information is available for this paper.
